# Appropriateness of Ketoanalogues of Amino Acids, Calcium Citrate, and Inulin Supplementation for CKD Management: A RAND/UCLA Consensus

**DOI:** 10.3390/nu16172930

**Published:** 2024-09-02

**Authors:** Nadia Saavedra-Fuentes, Enrique Carmona-Montesinos, Gilberto Castañeda-Hernández, Israel Campos, Juan Carlos Castillo-Salinas, Javier Alberto Castillo-Tapia, Karla Guadalupe Del Castillo-Loreto, Juan Carlos Falcón-Martínez, Raquel Fuentes-García, Miguel Ángel García de León Guerrero, Victor García-García, Erika F. Gómez-García, Rafael González-Toledo, Angélica Jaime, Kely Rely, Claudia Lerma, Luis E. Morales-Buenrostro, Mateo Quilantan-Rodriguez, Adrián Rodriguez-Matías, Felipe Octavio Rojas-Rodriguez, Rafael Valdez-Ortiz, Michael Wasung, Berenice Ceron-Trujillo, Edgar Ramirez-Ramirez

**Affiliations:** 1 Centro Medico Dalinde, Mexico City 06760, Mexico; drajaimesolis@gmail.com; 2 Hospital Trinidad, Mexico City 06760, Mexico; ecarmona@endocrinoroma.com; 3 Pharmacology Department, Centro de Investigación y de Estudios Avanzados del Instituto Politécnico Nacional, Mexico City 07360, Mexico; gcastane@cinvestav.mx; 4 Hospital General Dr. Miguel Silva, Morelia 58253, Mexico; israel.campos@gmx.com; 5 Independent Researcher, Mexico City 03010, Mexico; juancarloscassal@gmail.com; 6 Hospital General Regional 1 Vicente Guerrero, Acapulco 39616, Mexico; castapia76@hotmail.com; 7 Independent Researcher, Mexico City 06760, Mexico; karla.delcastillo9@hotmail.com; 8 MedSci Institute, Mexico City 10900, Mexico; jc.falconm@gmail.com; 9 Hospital General Regional 2 Dr. Guillermo Fajardo Ortiz, Mexico City 04929, Mexico; raquel.n.fuentes@gmail.com; 10 LAPI Unidad de Hemodiálisis, Naucalpan 53100, Mexico; nefrologogarcia@yahoo.com.mx; 11 Nephrology and Transplantation Unit, Unidad Medica de Alta Especialidad 14, Veracruz 91810, Mexico; wictor30@hotmail.com; 12 Medicine and Psychology Department, Universidad Autónoma de Baja California, Tijuana 22390, Mexico; erikaf.gomezg@gmail.com; 13 Independent Researcher, Metepec 52140, Mexico; rtoledo29@yahoo.com.mx; 14 Cost Effectiveness Assessment for Health Technology, Mexico City 09450, Mexico; rkely18@gmail.com; 15 Instituto Nacional de Cardiología Ignacio Chavez, Mexico City 14080, Mexico; dr.claudialerma@gmail.com; 16 Nephrology and Mineral Metabolism Department, Instituto Nacional de Ciencias Médicas y Nutrición Salvador Zubirán, Mexico City 14080, Mexico; luis_buenrostro@yahoo.com; 17 Centro Medico Renal de Tamaulipas, Ciudad Madero 89490, Mexico; drquilantanr@gmail.com; 18 Hospital Angeles Metropolitano, Mexico City 06760, Mexico; adrianrodmati@hotmail.com; 19 Hospital Mac La Viga, Mexico City 09430, Mexico; octavio.medicina@gmail.com; 20 Hospital General de Mexico, Mexico City 06720, Mexico; rafavaldez@gmail.com; 21 Hospital Angeles Lomas, Huixquilican 52763, Mexico; mwasung@gmail.com; 22 Aequitas Medica, Mexico City 03810, Mexico; berenice.ceron@aequitasmedica.com (B.C.-T.); edgar.ramirez@aequitasmedica.com (E.R.-R.)

**Keywords:** chronic kidney disease supplementation, consensus, renal outcomes, chronic kidney disease manifestations

## Abstract

Background: Current treatment for chronic kidney disease (CKD) focuses on improving manifestations and delaying progression. Nutritional approaches play a crucial role in CKD management, and various supplements have become available. Ketoanalogues of amino acids (KAs), calcium citrate, and inulin have been proposed as suitable supplements, yet their widespread use has been limited due to insufficient evidence. This study aimed to generate general guidance statements on the appropriateness of these supplements through a RAND/UCLA consensus process. Methods: A RAND/UCLA consensus panel was convened to evaluate the appropriateness of these supplements in different clinical scenarios. In this study, we present a subgroup analysis focusing on a panel of eleven clinical nephrologists from among the experts. Results: Supplementation of low-protein diets (LPDs) and very low-protein diets (VLPDs) with KA was considered appropriate to reduce manifestations and delay CKD outcomes, supplementation with calcium citrate is considered appropriate to reduce CKD manifestations, and supplementation with inulin is considered appropriate to delay CKD outcomes and manage comorbidities. Conclusions: Based on a combination of clinical experience and scientific evidence, the panel reached a consensus that KA supplementation of LPD and VLPD, calcium citrate, and inulin are appropriate in patients with CKD across various scenarios.

## 1. Introduction

Chronic kidney disease (CKD) is currently recognized as a significant contributor to global morbidity and mortality [1]. A study on global access to CKD treatment estimated that the number of people requiring renal replacement therapy (RRT) is expected to rise to 5.4 million persons by 2030 [2]. This is particularly worrisome as estimates indicate that between 2.2 and 7 million people died prematurely in 2010 worldwide due to poor accessibility to treatment, with most of these deaths occurring in low-income and middle-income countries (LICs and MICs, respectively) [2]. Since the 1990s, there has been a global trend shifting CKD mortality toward older ages; however, the opposite has been observed in a handful of countries, namely American Samoa, Guam, and Mexico [3]. Rising prevalence of diabetes and hypertension, coupled with limited access to RRT, are the main contributors to the increasing CKD burden in these locations [3].

As CKD is an irreversible disease, current treatment focuses on strategies aiming to improve the manifestations and outcomes of CKD [4]. Manifestations include CKD-mineral bone disorder (CKD-MBD) and acidosis, while outcomes encompass events related to disease progression, such as hospitalizations, major adverse cardiovascular events (MACEs), or initiation of RRT [4]. Intestinal dysbiosis plays a bidirectional role in disease progression [5].

Specialized nutritional care is essential in CKD patients, as nutritional requirements significantly change along the course of the disease, rendering them vulnerable to nutritional abnormalities [6]. For this reason, it is recommended that nutritional supplementation be tailored according to patients’ needs [6]. While there is extensive guidance with graded evidence for supplementation with certain nutrients, recommendations for low-protein diets (LPDs) and very low-protein diets (VLPDs) with ketoanalogues of amino acids (KAs), calcium citrate, and inulin are limited or absent in guidelines. KA, calcium citrate, and inulin are widely available over-the-counter supplements. Recently, a fixed-dose combination comprising these three components has become available in Mexico (Cetolán^®^), offering the added benefit of reducing pill burden.

Guideline recommendations require high-grade evidence, which may not be available for all these approaches. In routine practice, clinicians often face situations not addressed by guidelines, and evidence supporting the benefit of specific interventions is limited. Despite this, clinicians must still decide whether to recommend any of these supplementation strategies. To provide general guidance in these low-evidence scenarios, we aimed to establish the position of an expert group using the RAND Corporation/University of California, Los Angeles (RAND/UCLA) consensus methodology. This approach leverages clinicians’ expertise in situations where evidence is scarce. In a previous report, we presented an expert consensus focused on a wide range of nutritional approaches [7]. For this study, we focused our analysis on a subgroup of clinical nephrologists and narrowed our scope to these three supplements.

## 2. Materials and Methods

### 2.1. RAND/UCLA Appropriateness Method

**Figure 1:**
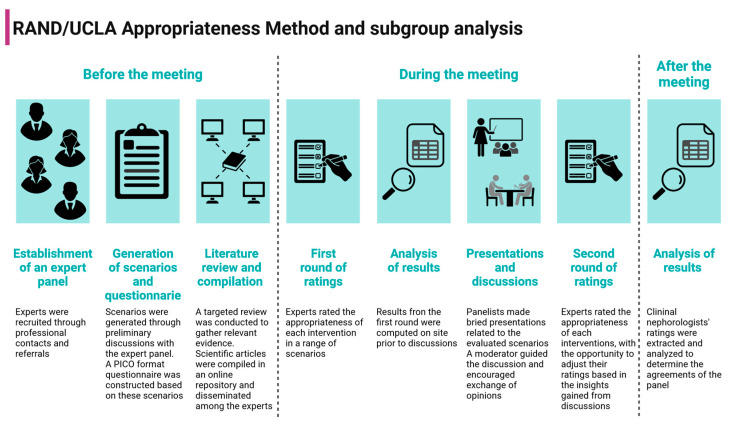
Diagram of the RAND/UCLA Appropriateness Method and subgroup analysis. Created with BioRender.com.

The RAND/UCLA Appropriateness Method is a formal consensus process in which experts form a group opinion on the “appropriateness” of an intervention based on a combination of available evidence and their clinical expertise. The appropriateness of an approach is determined by whether the expected health benefits outweigh the potential negative consequences by a significant margin to justify a procedure, regardless of cost [8]. This method is a validated, systematic approach for evaluating the appropriateness of interventions at a patient-specific level, particularly in cases where clinical trials are not feasible for every scenario. It has been used to a wide range of interventions [9,10,11,12,13,14]. A diagram of the RAND/UCLA Appropriateness Method and subgroup analysis is depicted in Figure 1.

### 2.2. Establishment of an Expert Panel

In our previous study, we assembled an expert panel comprising clinical nephrologists, along with clinicians from other specialties, nutritionists, pharmacologists, and clinical researchers [7]. The experts were recruited through professional contacts and referrals, with eligibility criteria mandating a minimum of five years of experience in managing CKD patients; those barred from clinical practice were excluded [7]. Compensation for their time was provided by Laboratorios Columbia Comercial, though it is important to note that the sponsor had no influence over the study’s methodology, results, or discussions. For this particular study, we specifically selected and analyzed the responses from clinical nephrologists within the panel.

### 2.3. Generation of Scenarios and Literature Review

We developed a list of scenarios outlining a selection of nutritional approaches pertinent for patients with CKD. A draft of scenarios and categories was further refined through preliminary discussions with the expert panel. These scenarios were used to construct a questionnaire with individual questions in a Population, Intervention, Comparison, Outcome format [7].

Using these scenarios, we conducted a targeted literature review to gather relevant evidence. Scientific articles were compiled in an online repository and disseminated among the expert panel members prior to the rating rounds. The literature review was conducted in January 2024 using PubMed and employing the following MESH terms: “Renal Insufficiency, Chronic*” OR “Chronic Kidney Disease” AND “Dietary Supplements” OR “Amino Acids” OR “Keto Acids” OR “Calcium Citrate/pharmacology*” OR “Inulin/therapeutic use” AND “Renal Dialysis” OR “Medication Adherence*” OR “Dysbiosis” OR “Uremic Toxins”. Due to the scarcity of scientific articles related to our search, a systematic review approach was deemed unfeasible. Panel members provided literature they deemed relevant, supplementing the review process [7]. In our previous study, experts rated scenarios stratified along 5 categories; for this study, we focused on three of the five original categories related to the glomerular filtration rate (GFR): Grade 3a (GFR 45–59 mL/min/1.73 m^2^), Grades 3b and 4 (GFR 15–44 mL/min/1.73 m^2^), and Grade 5 (GFR < 15 mL/min/1.73 m^2^) [7].

### 2.4. Two-Round Consensus

Panelists gathered for a face-to-face meeting in March 2024 in Mexico City. During this event, members of the expert panel completed the first round of the questionnaire rating each scenario in a 1 to 9 scale with “1” being “highly inappropriate” and “9” being “highly appropriate”. Results were computed in real time on site. Following the first round, panelists made brief presentations on relevant literature to selected scenarios. After each presentation, a moderator facilitated a discussion to explore expert insights, encouraging broad participation and exchange of contrasting opinions. Subsequently, a second round of ratings was conducted. In this round, experts received a similar questionnaire that included their previous responses as well as ratings from the rest of the panel. Panel members were given the opportunity to maintain their initial ratings or adjust them based on the insights gained from the presentations and discussions [7].

### 2.5. Statistical Analysis

The results of clinical nephrologists’ ratings were entered into a spreadsheet in Microsoft Excel (Redmond, WA, USA: Microsoft, v2407), manually verified and then exported to a Tab-Separated Values format. Physical copies of questionnaires were stored, and the data were independently verified twice by two different persons. To analyze the data, the median and the disagreement index (DI) of each scenario were calculated using a Python (Python Language Reference, version 3.12. Wilmington, DE, USA: Python Software Foundation, 2023) script following RAND Corporation (Santa Monica, CA, USA: RAND, 2001) guidelines [8]. Agreement was considered achieved when the DI was less than 1. The Python script used for these calculations is publicly available in a GitHub repository (https://github.com/DrEdgarRamirez/AequitasMedica, accessed on 19 March 2024).

### 2.6. Ethical Compliance

No bioethical committee approval was required due to the nature of this study.

## 3. Results

### 3.1. Summary of Participants and Answers

**Figure 2:**
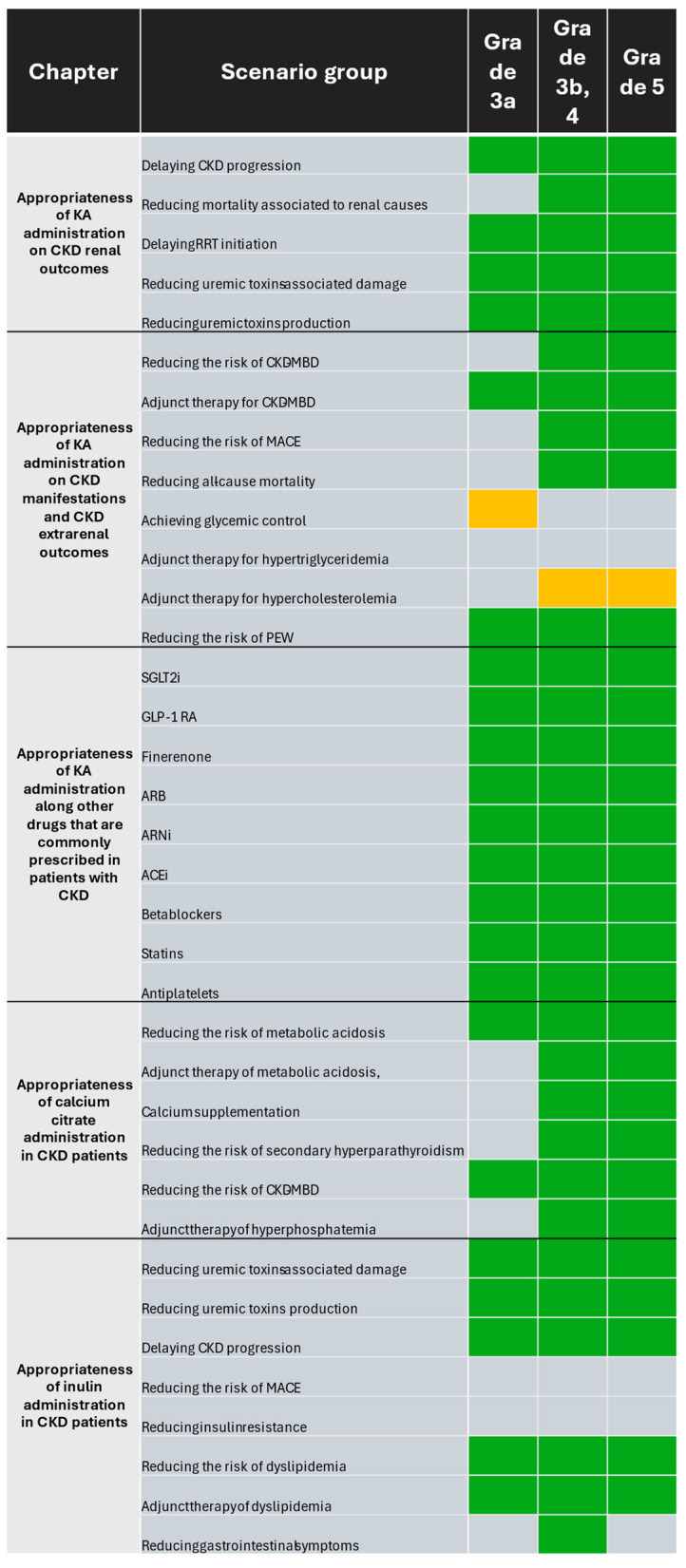
Appropriateness of supplementation with KA, calcium citrate, and inulin supplementation in CKD patients according to GFR grade. Scenarios where agreement was reached and median of ratings was between 4 and 6 were classified as uncertain (amber background), whereas those with a median score between 7 and 9 were classified as appropriate (green background). Agreement was not reached for scenarios where DI ≥ 1 (gray background). Abbreviations: ACEi = angiotensin-converting enzyme inhibitor; ARB = angiotensin II receptor blocker; ARNi = angiotensin receptor neprilysin inhibitor; CKD = chronic kidney disease; CKD-MBD = chronic kidney disease mineral bone disorder; GLP-1 RA = glucagon-like peptide 1 receptor agonist; KA = ketoanalogue of amino acids; RRT = renal replacement therapy; MACE = major adverse cardiovascular event; PEW = protein-energy wasting; SGLT2i = sodium glucose co-transporter type 2 inhibitor.

Eleven nephrologists participated in the expert panel. Of them, six (55%) had a primary role in a public institution, whereas five (45%) worked at private institutions as their primary role. Collectively, experts answered 1148 of 1188 questions for a 96% answer rate. Consensus was reached for 87 of 108 scenarios (80%). A summary of the consensus results is depicted in Figure 2.

### 3.2. Appropriateness of Ketoanalogues of Amino Acid Supplementation on Chronic Kidney Disease Renal Outcomes

Panelists rated five outcomes with three categories each for a total of 15 scenarios. Consensus was reached in 14 scenarios (93%) comprising five outcomes. The panelists considered the supplementation of LPD and VLPD with KA appropriate in patients with CKD for delaying CKD progression (grades 3a-5, DI < 1 in each scenario) and RRT initiation (grades 3a-5, DI < 1 in each scenario), reducing mortality associated with renal causes (grades 3b-5, DI < 1 in each scenario), and reducing uremic toxin production (grades 3a-5, DI < 1 in each scenario) and their associated damage (grades 3a-5, DI < 1 in each scenario). Individual ratings, median, and DI values are available in Table S1.

### 3.3. Appropriateness of Ketoanalogues of Amino Acid Supplementation on Chronic Kidney Disease Manifestations and Extrarenal Outcomes

Panelists rated eight manifestations and extrarenal outcomes with three categories each for a total of 24 scenarios. Consensus was reached in 15 scenarios (62%) for which 12 scenarios received “appropriate” ratings (50%). KA supplementation of LPD and VLPD was considered appropriate for both reducing the risk of CKD-MBD (grades 3b-5, DI < 1 in each scenario) and as adjunct therapy for CKD-MBD (grades 3a-5, DI < 1 in each scenario), as well as reducing the risk of MACE (grades 3b-5, DI < 1 in each scenario) and PEW (grades 3a-5, DI < 1 in each scenario) and reducing all-cause mortality (grades 3b-5, DI < 1 in each scenario). Individual ratings, median, and DI values are available in Table S1.

### 3.4. Appropriateness of Ketoanalogues of Amino Acid Supplementation along with Other Drugs That Are Commonly Prescribed in Patients with Chronic Kidney Disease

Panelists rated combining the supplementation of LPD and VLPD with KA with nine drugs that are commonly prescribed to patients with CKD, grouped into three categories each, for a total of 27 scenarios. Consensus was reached in all 27 scenarios (100%) with all of them rated as “appropriate”. The drugs for which the supplementation of LPD and VLPD with KA was considered appropriate for concomitant use included sodium glucose co-transporter type 2 inhibitors (SGLT2i) (grades 3a-5, DI < 1 in each scenario), glucagon-like peptide 1 receptor agonists (GLP-1 RA) (grades 3a-5, DI < 1 in each scenario), finerenone (grades 3a-5, DI < 1 in each scenario), angiotensin II receptor blockers (ARBs) (grades 3a-5, DI < 1 in each scenario), angiotensin receptor neprilysin inhibitors (ARNi) (grades 3a-5, DI < 1 in each scenario), angiotensin-converting enzyme inhibitors (ACEi) (grades 3a-5, DI < 1 in each scenario), beta-blockers (grades 3a-5, DI < 1 in each scenario), statins (grades 3a-5, DI < 1 in each scenario), and antiplatelets (grades 3a-5, DI < 1 in each scenario). Individual ratings, median, and DI values are available in Table S1.

### 3.5. Appropriateness of Calcium Citrate Supplementation in Chronic Kidney Disease Patients

Panelists evaluated eighteen scenarios of calcium citrate supplementation across six scenario groups. Consensus was reached in fourteen scenarios (77%), with all ratings categorized as “appropriate”. Calcium citrate supplementation was considered appropriate for both reducing the risk (grades 3a-5, DI < 1 in each scenario) and as adjunct therapy (grades 3b-5, DI < 1 in each scenario) of metabolic acidosis; it was also considered appropriate for reducing the risk of CKD-MBD (grades 3a-5, DI < 1 in each scenario) and secondary hyperparathyroidism (grades 3b-5, DI < 1 in each scenario), as well as adjunct therapy of hyperphosphatemia (grades 3b-5, DI < 1 in each scenario) and as a calcium supplement (grades 3b-5, DI < 1 in each scenario). Individual ratings, median, and DI values are available in Table S1.

### 3.6. Appropriateness of Inulin Supplementation in Chronic Kidney Disease Patients

Panelists evaluated twenty-four scenarios of inulin supplementation across eight scenario groups. Consensus was reached in 17 scenarios (70%), with all of them rated as “appropriate”. Inulin supplementation was deemed appropriate for reducing uremic toxin production (grades 3a-5, DI < 1 in each scenario) and their associated damage (grades 3a-5, DI < 1 in each scenario), both reducing the risk (grades 3a-5, DI < 1 in each scenario) and as adjunct therapy of dyslipidemia (grades 3a-5, DI < 1 in each scenario), and it was also considered appropriate for delaying CKD progression (grades 3a-5, DI < 1 in each scenario) and reducing gastrointestinal symptoms (grades 3b-5, DI < 1 in each scenario). Individual ratings, median, and DI values are available in Table S1.

## 4. Discussion

Patients with CKD are particularly susceptible to nutritional abnormalities, which significantly increase the risks of morbidity, disease progression, and mortality. Consequently, guidelines recommend nutritional therapy with appropriate supplementation to mitigate comorbid conditions, reduce manifestations, and delay disease progression and adverse outcomes [6]. Given the inherent limitations of guidelines to evaluate every possible nutritional strategy, we aimed to establish an expert consensus on the appropriateness of KA supplementation to LPD and VLPD, as well as calcium citrate and inulin supplementation for CKD patients.

### 4.1. Supplementation of Low-Protein Diets and Very Low-Protein Diets with Ketoanalogues of Amino Acids Is Considered Appropriate to Reduce Manifestations and Delay Outcomes in Chronic Kidney Disease Patients

Excessive protein intake significantly contributes to the progression of CKD [4]. Since proteins cannot be stored in the body, any excess intake must be catabolized, resulting in the production of uremic toxins [4]. As CKD progresses, these byproducts accumulate, leading to organ dysfunction [4]. Thus, LPD and VLPD have a long-standing history of use to alleviate clinical symptoms and delay the need for RRT [4]. However, protein intake restriction carries an inherent risk of essential amino acid deficiency [4]. To mitigate this risk, supplementation with KA is commonly recommended. Since KAs lack the amino group, they serve as substrates for protein synthesis without increasing the nitrogen load [4]. Additionally, dietary protein restriction induces afferent arteriole vasoconstriction, reducing intraglomerular hypertension and further contributing to the delay of CKD progression [15].

Uremic toxins exert a diverse range of harmful effects on renal function, including the generation of reactive oxygen species (ROS), tissue inflammation and fibrosis, epithelial–mesenchymal transition, activation of the renin–angiotensin system, intracellular toxin accumulation, reduction in peritubular capillaries, and shortening of telomeres. These mechanisms collectively contribute to nephrotoxicity and accelerate disease progression [16].

Our expert panel agreed that KA supplementation of LPD or VLPD is appropriate for delaying CKD progression and RRT initiation. Two meta-analyses, one conducted in 2016 that included 9 studies, and another in 2019 of 13 studies, both concluded that KA supplementation significantly reduces the estimated glomerular filtration rate (eGFR) decline among CKD patients, regardless of whether they were following LPD or VLPD regimes [17,18]. Moreover, three recent studies have demonstrated the efficacy of KA supplementation of LPD or VLPD to delay RRT initiation [19,20,21].

The panel agreed that KA-supplemented LPD or VLPD is appropriate for reducing mortality associated with renal causes. In a retrospective study of 129 patients conducted in 2023, this approach was associated with a protective effect in terms of mortality due to renal causes [22]. While several studies may present mixed results, a recent pool of four studies concluded that KA supplementation reduces risk of renal death by 35% in CKD stages 3–5 [6].

Our panel agreed that supplementing LPD and VLPD with KA is appropriate for reducing uremic toxin production and its associated damage. This strategy helps decrease the generation of nitrogen metabolism byproducts, thereby protecting patients from uremic toxicity [23]. Furthermore, a 2013 clinical trial demonstrated that a KA-supplemented VLPD intervention resulted in a significant reduction in serum concentrations of uremic toxins by 36% within the first week of treatment [24].

The panel agreed that KA supplementation is appropriate for reducing the risk of CKD-MBD and as adjunct therapy for this manifestation. Results of the metanalysis of nine studies comprising over 410 patients concluded that KA supplementation of VLPD ameliorated CKD-MBD, as it was associated with lower phosphate, PTH (parathyroid hormone), and FGF23 levels, while maintaining serum calcium levels [18]. Furthermore, results of a 2019 metanalysis of twelve studies concluded that early KA intervention is effective for reversing CKD-MBD in severe cases [17].

Panelists concluded that KA supplementation of LPD or VLPD is appropriate for reducing the risk of MACE and PEW. In a cohort study involving 15,782 patients, KA supplementation was associated with significant reductions in coronary artery disease (CAD) events by 29%, stroke by 32%, and MACE by 24% [19]. Additionally, a retrospective study of 3282 patients showed that KA supplementation was associated with a 39% lower risk of MACE within the first year of treatment compared with patients who did not receive KA supplementation [19]. KA supplementation prevents muscle degradation by serving as precursors of amino acids, thus supporting protein synthesis, especially in patients following LPD or VLPD [25]. Importantly, KAs also help reduce nitrogen and acid loads in these patients, contributing to the management of their kidney disease and overall metabolic balance [25].

Panelists agreed that KA supplementation in patients undergoing LPD or VLPD is appropriate for reducing all-cause mortality. In a 2021 cohort study, patients supplemented with KA in addition to following LPD or VLPD displayed a 27% lower 5-year all-cause mortality rate compared with those without KA supplementation [20]. Additionally, a 2017 cohort reported a 51% reduction in the composite of all-cause death and initiation of RRT among patients receiving KA supplementation [21]. Furthermore, a pharmacoeconomic study conducted in 2023 demonstrated that KA supplementation extended survival in CKD patients by an average of 1.22 years [26].

The expert panel reached a consensus that combining KA-supplemented LPD or VLPD with various pharmacological treatments is appropriate. These treatments include SGLT2i, GLP-1 RA, finerenone, ARB, ACEi, ARNi, beta-blockers, statins, and antiplatelets. Current recommendations for delaying CKD progression feature SGLT2i, GLP-1 RA, finerenone, ARB, and ACEi. ARNi and beta-blockers are recommended for patients with CKD and heart failure, while statins and antiplatelets are advised for those with CKD and cardiovascular disease [4,27]. To the best of our knowledge, no clinical trials have investigated the benefits of combining these drugs with KA-supplemented LPD or VLPD. However, a potential synergistic mechanism with ACEi, ARB, or SGLT2i has been proposed due to their complementary mechanisms of action, and no deleterious interactions have been demonstrated [4,28]. Additionally, the acid-sparing effect of LPD and VLPD can potentially amplify the renal protective effects of these drugs, though clinical trials are required to confirm these theoretical benefits [4,28].

### 4.2. Supplementation with Calcium Citrate Is Considered Appropriate to Reduce Manifestations of Chronic Kidney Disease

Calcium citrate is frequently used as a dietary source of calcium for patients who do not reach the recommended intake [29]. In advanced CKD, there is a decrease in calcium concentration and intestinal absorption, which contributes to the development of secondary hyperparathyroidism and bone disorders [6]. Due to these factors, calcium supplementation is recommended in CKD patients [6]. Additionally, citrate acts as an alkalinizing agent [30].

Our expert panel agreed that calcium citrate supplementation is appropriate for reducing the risk of metabolic acidosis and as adjunct therapy for this condition. Metabolic acidosis is common among CKD patients and contributes to muscle wasting, bone demineralization, and tubulointerstitial fibrosis, all of which accelerate CKD progression [30,31]. Multiple lines of experimental evidence support the alkalinizing effect of calcium citrate, which helps ameliorate metabolic acidosis [30,32,33].

The panel of experts agreed that calcium citrate supplementation is appropriate for reducing the risk of CKD-MBD, secondary hyperparathyroidism, and as adjunct therapy for hyperphosphatemia. Calcium citrate improves serum phosphorus levels and reduces PTH levels up to four times more effectively than calcium carbonate, resulting in a significant improvement in overall bone density [29,33,34,35]. Additionally, the panel considered calcium citrate to be an appropriate agent for calcium supplementation due to its superior bioavailability, which results in a 55% greater increase in serum calcium levels compared with calcium carbonate [29,33,35].

### 4.3. Supplementation with Inulin Is Considered Appropriate to Delay Chronic Kidney Disease Outcomes and Manage Comorbidities

Gut dysbiosis results in elevated urea levels in the digestive system, intensifying the circulation of uremic toxins, while CKD contributes to changes in microbiome composition, thereby establishing a bidirectional relationship [5,36]. Uremic toxins are implicated not only in CKD progression but also in cardiovascular disease morbidity and mortality [5]. Consequently, therapeutic strategies aimed at reducing uremic toxins have been proposed as therapeutic alternatives [5]. Moreover, certain uremic toxins are inadequately eliminated by dialysis, which has sparked the interest in strategies aimed at restoring the gut microbiome to improve outcomes in CKD patients [36].

Our expert panel agreed that inulin supplementation is appropriate for reducing the production and damage caused by uremic toxins, as well as for delaying CKD progression. Clinical trials have demonstrated the benefits of inulin supplementation in reducing uremic toxins in CKD patients, which may prove useful in delaying disease progression [37,38].

The panel concluded that it is appropriate to supplement CKD patients with inulin for reducing the risk of dyslipidemia, and as an adjunct therapy, as well as for reducing gastrointestinal symptoms. In CKD patients, inulin supplementation has been associated with metabolic benefits, including reducing total cholesterol and triglycerides, as well as increasing HDL cholesterol levels [38]. Prebiotics, such as inulin, exhibit a low incidence of gastrointestinal side effects in CKD patients, suggesting potential benefits in symptom management [39]. However, clinical trials investigating this issue are necessary to confirm their potential therapeutic benefit.

### 4.4. Future Perspectives

The RAND/UCLA consensus methodology relies on expert opinion and clinical expertise in low evidence settings through guided discussions in face-to-face environments. It acknowledges that clinicians often encounter situations with scarce scientific literature yet must make diagnostic or therapeutic decisions. The scenarios portrayed in this consensus represent some of the real-world challenges they face in clinical practice. Although these approaches may not align with recommendations in guidelines based on graded evidence, the expert group concurs that available evidence consistently supports the benefits of these interventions for CKD patients. Further clinical trials are expected to address these gaps in knowledge.

Supplementing LPD and VLPD with KA, calcium citrate, and inulin may be a suitable population-wide intervention in CKD patients in order to ease the burden on healthcare systems in LIC and MIC, particularly where access to RRT is limited. A few pharmacoeconomic studies on KA supplementation have demonstrated that this approach is effective for delaying CKD progression and RRT initiation while also reducing costs [22,26]. Further studies focusing on the economic advantages of calcium citrate and inulin supplementation in CKD patients, as well as a broader application of KA-supplemented LPD or VLPD, are needed to clarify these benefits.

### 4.5. Strengths and Limitations

Our study was strengthened by fostering interactions among experts through the establishment of working groups and presentations, which facilitated deeper insights and interactions within the panel. We consider this strategy particularly enriching for our discussions, as the RAND/UCLA methodology derives significant advantage from face-to-face interactions and clinical expertise.

Our study is not without limitations. Given the limited evidence, we acknowledge that some studies may be outdated or underpowered by contemporary standards. Future studies, including large clinical trials and systematic reviews with meta-analysis, are required to obtain high-quality evidence and generate evidence-based guidelines. In such cases, we particularly adhered to a risk–benefit evaluation based on the best available evidence and clinical expertise. Our consensus is limited to only three supplementation strategies. While we recognize that they do not represent every nutritional approach for CKD patients, they include some of the most widely available supplements with clear evidence supporting their benefits in CKD management.

## 5. Conclusions

Our panel reached a consensus that KA supplementation of LPD and VLPD, calcium citrate, and inulin are appropriate in patients with CKD in a range of scenarios. This consensus is based on a combination of clinical experience and scientific evidence. Future clinical trials and meta-analyses are necessary to evaluate the impact of these strategies on the outcomes of CKD patients.

## Data Availability

The original contributions presented in the study are included in the article/Supplementary Materials, further inquiries can be directed to the corresponding author.

## Supplementary Materials

The following supporting information can be downloaded at: https://www.mdpi.com/article/10.3390/nu16172930/s1, Table S1: Median and DI of all scenarios, stratified according to CKD category.
