# Supplementary material for: Appropriateness of Ketoanalogues of Amino Acids, Calcium Citrate, and Inulin Supplementation for CKD Management: A RAND/UCLA Consensus
Source: Nutrients. 2024 Sep 2;16(17):2930. doi: 10.3390/nu16172930 (PMC11397001; doi:10.3390/nu16172930)
Supplement: Supplementary file 1 [file nutrients-16-02930-s001.zip › nutrients-3174664-supplementary.pdf]

Supplementary Material

Appropriateness of Ketoanalogues of Amino Acids, Calcium Citrate, and Inulin Supplementation for CKD Management: A RAND/UCLA Consensus

Chapter	Scenario Group	Category (CKD Grade)	Panelist Ratings											Median	DI
Appropriateness of KA administration on CKD renal outcomes	Delaying CKD progression	3a	9	9	8	1	6	9	8	9	7	9	8	**8**	**0.49180328**
		3b, 4	9	9	9	7	8	9	9	9	9	9	8	**9**	**0.13157895**
		5	9	9	9	7	8	9	9	9	9	9	8	**9**	**0.13157895**
	Reducing mortality associated to renal causes	3a	7	9	9	1	8	9	8	8	5	7	3	8	1.55844156
		3b, 4	8	9	9	7	8	9	9	9	8	7	3	**8**	**0.2919708**
		5	7	9	9	5	8	9	9	9	9	7	3	**9**	**0.74766355**
	Delaying RRT initiation	3a	9	9	9	1	8	9	9	9	9	9	7	**9**	**0.2919708**
		3b, 4	9	9	9	7	8	9	9	9	9	9	7	**9**	**0.2919708**
		5	9	9	9	5	8	9	9	9	9	9	7	**9**	**0.2919708**
	Reducing uremic toxins-associated damage	3a	8	9	9	1	7	9	9	7	5	8	-	**8**	**0.87128713**
		3b, 4	9	9	9	6	7	9	9	9	9	9	-	**9**	**0.3099631**
		5	9	9	9	6	7	9	9	9	9	9	-	**9**	**0.3099631**
	Reducing uremic toxins production	3a	9	9	9	1	8	9	8	8	5	8	-	**8**	**0.87128713**
		3b, 4	9	9	9	7	8	9	9	9	9	9	-	**9**	**0.1461794**
		5	9	9	9	7	8	9	9	9	9	9	-	**9**	**0.1461794**
Appropriateness of KA administration on CKD manifestations and CKD extrarenal outcomes	Reducing the risk of CKD-MBD	3a	8	9	8	1	3	9	9	8	5	6	5	8	1.55844156
		3b, 4	8	9	8	7	5	9	7	8	7	7	6	**7**	**0.49180328**
		5	8	9	8	7	5	9	8	9	7	7	6	**8**	**0.49180328**
	Adjunct therapy for CKD-MBD	3a	6	9	9	1	6	9	8	9	5	7	6	**7**	**0.74766355**
		3b, 4	9	9	8	7	6	9	8	8	9	8	6	**8**	**0.49180328**
		5	9	9	8	7	6	9	8	8	9	8	6	**8**	**0.49180328**
	Reducing the risk of MACE	3a	7	9	7	1	3	9	7	7	7	7	8	7	1.55844156
		3b, 4	7	9	8	6	5	9	7	9	7	7	8	**7**	**0.49180328**
		5	9	9	8	6	5	9	7	9	9	7	8	**8**	**0.49180328**
	Reducing all-cause mortality	3a	5	9	8	1	5	9	6	8	5	4	6	6	1.08695652
		3b, 4	5	9	8	5	7	9	9	9	8	4	6	**8**	**0.74766355**
		5	5	9	8	5	7	9	8	9	8	4	6	**8**	**0.74766355**
	Achieving glycemic control	3a	4	7	4	1	5	9	5	7	5	4	5	5	**0.96774194**
		3b, 4	4	9	4	1	5	9	5	8	8	4	5	5	1.08695652
		5	4	9	4	1	5	9	5	8	8	4	5	5	1.08695652
	Adjunct therapy for hypertriglyceridemia	3a	5	9	3	1	5	9	6	6	9	4	5	5	1.55844156
		3b, 4	8	9	3	6	5	9	6	8	9	4	5	6	1.08695652
		5	5	9	3	6	5	9	6	8	9	4	5	6	1.08695652
	Adjunct therapy for hypercholesterolemia	3a	5	9	-	1	5	6	6	6	1	4	6	5.5	1.21142857
		3b, 4	6	9	-	6	5	6	6	6	8	4	6	6	**0.69565217**
		5	6	9	-	6	5	6	6	6	8	4	6	6	**0.69565217**
	Reducing the risk of PEW	3a	6	9	9	1	7	9	9	8	5	9	-	**8.5**	**0.87128713**
		3b, 4	6	9	9	7	7	9	9	9	9	9	-	**9**	**0.3099631**
		5	6	9	9	7	7	9	9	9	9	9	-	**9**	**0.3099631**

Appropriateness of KA administration along other drugs that are commonly prescribed in patients with CKD	SGLT2i	3a	9	9	9	1	8	9	9	9	9	7	8	**9**	**0.2919708**
		3b, 4	9	9	9	5	8	9	9	9	9	9	8	**9**	**0.13157895**
		5	9	9	9	5	8	9	9	9	9	9	8	**9**	**0.13157895**
	GLP-1 RA	3a	9	9	9	1	5	9	9	9	9	7	8	**9**	**0.74766355**
		3b, 4	9	9	9	5	8	9	9	9	9	9	8	**9**	**0.13157895**
		5	9	9	9	5	8	9	9	8	9	9	8	**9**	**0.13157895**
	Finerenone	3a	9	9	9	1	5	9	9	8	9	5	8	**9**	**0.74766355**
		3b, 4	9	9	9	5	8	9	9	8	9	9	8	**9**	**0.13157895**
		5	9	9	9	5	8	9	9	8	9	9	8	**9**	**0.13157895**
	ARB	3a	9	9	9	1	7	9	9	8	5	7	8	**8**	**0.74766355**
		3b, 4	9	9	9	5	7	9	9	9	9	9	8	**9**	**0.2919708**
		5	9	9	9	5	7	9	9	9	9	9	8	**9**	**0.2919708**
	ARNi	3a	9	9	9	1	8	9	9	9	9	7	8	**9**	**0.2919708**
		3b, 4	9	9	9	5	8	9	9	9	9	9	8	**9**	**0.13157895**
		5	9	9	9	5	8	9	9	8	9	9	8	**9**	**0.13157895**
	ACEi	3a	9	9	9	1	8	9	9	9	9	7	8	**9**	**0.2919708**
		3b, 4	9	9	9	5	8	9	9	9	9	9	8	**9**	**0.13157895**
		5	9	9	9	5	8	-	9	9	9	9	8	**9**	**0.17627119**
	Betablockers	3a	9	9	9	1	5	-	9	8	9	7	8	**8.5**	**0.87128713**
		3b, 4	9	9	9	5	7	-	9	9	9	9	8	**9**	**0.32835821**
		5	9	9	9	5	7	-	9	8	9	9	8	**9**	**0.32835821**
	Statins	3a	9	9	9	1	5	-	9	9	9	7	8	**9**	**0.87128713**
		3b, 4	9	9	9	5	7	-	9	9	9	9	8	**9**	**0.32835821**
		5	9	9	9	5	7	-	9	8	9	9	8	**9**	**0.32835821**
	Antiplatelets	3a	9	9	9	1	5	-	9	8	9	7	8	**8.5**	**0.87128713**
		3b, 4	9	9	9	5	8	-	9	8	9	9	8	**9**	**0.17627119**
		5	9	9	9	5	8	-	9	8	9	9	8	**9**	**0.17627119**
Appropriateness of calcium citrate administration in CKD patients	Reducing the risk of metabolic acidosis	3a	5	9	-	1	7	9	9	8	9	6	8	**8**	**0.87128713**
		3b, 4	6	9	-	7	7	9	9	9	9	7	8	**8.5**	**0.3099631**
		5	6	9	-	7	7	9	9	9	9	7	8	**8.5**	**0.3099631**
	Adjunct therapy of metabolic acidosis,	3a	6	9	-	1	7	9	4	7	7	7	5	7	1.21142857
		3b, 4	8	9	-	7	7	9	7	8	9	7	5	**7.5**	**0.32835821**
		5	8	9	-	7	7	9	7	9	9	7	5	**7.5**	**0.32835821**
	Calcium supplementation	3a	8	9	-	1	7	8	9	8	8	4	6	8	1.21142857
		3b, 4	8	9	-	6	7	9	9	8	9	7	6	**8**	**0.49180328**
		5	8	9	-	6	7	9	9	9	9	7	6	**8.5**	**0.49180328**
	Reducing the risk of secondary hyperparathyroidism	3a	4	9	-	1	7	9	9	9	5	3	5	6	1.67567568
		3b, 4	6	9	-	1	7	9	9	9	9	7	5	**8**	**0.87128713**
		5	6	9	-	1	7	9	9	9	9	7	5	**8**	**0.87128713**
	Reducing the risk of CKD-MBD	3a	7	9	-	1	7	9	9	7	5	6	6	**7**	**0.87128713**
		3b, 4	7	9	-	1	7	9	9	9	9	6	6	**8**	**0.61135371**
		5	7	9	-	1	7	9	9	9	9	6	6	**8**	**0.61135371**
	Adjunct therapy of hyperphosphatemia	3a	4	9	-	1	7	9	9	8	9	7	8	8	1.21142857
		3b, 4	7	9	-	1	7	9	9	8	9	7	8	**8**	**0.40625**
		5	7	9	-	1	7	9	9	9	9	7	8	**8.5**	**0.40625**
Appropriateness of inulin administration in CKD patients	Reducing uremic toxins-associated damage	3a	9	9	8	1	8	9	7	8	9	7	7	**8**	**0.2919708**
		3b, 4	9	9	8	7	8	9	7	9	9	8	7	**8**	**0.2919708**
		5	9	9	8	7	8	9	8	9	9	8	7	**8**	**0.2919708**
	Reducing uremic toxins production	3a	9	9	8	1	8	9	7	8	9	7	7	**8**	**0.2919708**
		3b, 4	9	9	9	7	8	9	9	9	9	9	7	**9**	**0.2919708**
		5	9	9	9	7	8	9	9	9	9	9	7	**9**	**0.2919708**

	Delaying CKD progression	3a	8	9	8	1	8	7	7	9	9	7	7	**8**	**0.2919708**
		3b, 4	9	9	8	7	8	7	7	9	9	8	7	**8**	**0.2919708**
		5	9	9	8	7	8	9	7	9	9	8	7	**8**	**0.2919708**
	Reducing the risk of MACE	3a	7	3	7	1	6	5	7	8	7	6	7	7	1.70212766
		3b, 4	8	3	7	4	7	7	7	8	7	6	7	7	1.03896104
		5	8	3	7	4	7	7	7	8	7	6	7	7	1.03896104
	Reducing insulin resistance	3a	9	3	8	1	8	8	8	8	9	7	7	8	1.55844156
		3b, 4	9	3	8	1	8	8	8	8	9	8	7	8	1.55844156
		5	9	3	8	1	8	8	8	8	9	9	7	8	1.55844156
	Reducing the risk of dyslipidemia	3a	9	9	7	1	7	7	7	8	9	6	8	**7**	**0.49180328**
		3b, 4	9	9	7	1	7	9	8	8	9	8	8	**8**	**0.2919708**
		5	9	9	7	1	7	9	9	8	9	8	8	**8**	**0.2919708**
	Adjunct therapy of dyslipidemia	3a	9	7	7	1	7	7	7	8	9	6	8	**7**	**0.49180328**
		3b, 4	9	7	7	1	7	7	7	8	9	8	8	**7**	**0.2919708**
		5	9	7	7	1	7	7	7	8	9	9	8	**7**	**0.2919708**
	Reducing gastrointestinal symptoms	3a	9	7	9	1	7	7	9	8	9	3	7	7	1.55844156
		3b, 4	9	7	9	7	7	7	9	8	9	5	7	**7**	**0.2919708**
		5	9	7	9	7	7	7	9	8	9	5	7	**7**	**0.291970803**

Table S1. Median and DI of all scenarios, stratified according to CKD category. The table shows all scenarios, which were grouped in "chapters". Consensus was reached when DI < 1 (in bold). Ratings were considered "appropriate" when median ≥ 7 (in bold when DI < 1). Abbreviations: *ACEi* = angiotensin-converting enzyme inhibitors; *ARB* = angiotensin II receptor blockers; *ARNi* = angiotensin receptor neprilysin inhibitors; *CKD* = chronic kidney disease; *CKD-MBD* = chronic kidney disease mineral bone disorder; *GLP-1 RA* = glucagon-like peptide 1 receptor agonists; *KA* = ketonanalogues of amino acids; *RRT* = renal replacement therapy; *MACE* = major adverse cardiovascular events; *PEW* = protein-energy wasting; *SGLT2i* = sodium glucose co-transporter type 2 inhibitors.
